# Encapsulation of Cochleates Derived from *Salmonella* Infantis with Biopolymers to Develop a Potential Oral Poultry Vaccine

**DOI:** 10.3390/polym13193426

**Published:** 2021-10-06

**Authors:** Constanza Avendaño, Sonia Vidal, María Gabriela Villamizar-Sarmiento, Miguel Guzmán, Héctor Hidalgo, Lisette Lapierre, Carolina Valenzuela, Leonardo Sáenz

**Affiliations:** 1 Faculty of Veterinary Sciences, University of Chile, Santiago 8820808, Chile; constanza.avendano@ug.uchile.cl (C.A.); svidal@vaccimed.cl (S.V.); mvillamizar@postqyf.uchile.cl (M.G.V.-S.); mguzmanm@udla.cl (M.G.); hhidalgo@uchile.cl (H.H.); llapierre@uchile.cl (L.L.); 2 Department of Sciences and Pharmaceutical Technology, University of Chile, Santiago 8380494, Chile; 3 Nucleus of Applied Research in Veterinary and Agronomic Sciences, NIAVA, Faculty of Veterinary Medicine and Agronomy, Universidad de las Américas, Santiago 9250000, Chile

**Keywords:** cochleate, encapsulation, poultry, *Salmonella* Infantis, vaccine

## Abstract

The aim of this study was to develop and characterize *Salmonella* *enterica* serovar Infantis (*S.* Infantis) cochleates protected by encapsulation technology as a potential vaccine and to determine its safety in pullets. Cochleates were encapsulated by two technologies, spray drying and ionotropic gelation at different concentrations (0–15% v/v), and were characterized by physicochemical properties, protein content and Fourier Transform Infrared Spectroscopy (FTIR). The cochleates were white liquid suspensions with tubular shapes and a protein content of 1.0–2.1 mg/mL. After encapsulation by spray drying, microparticles ranged in size from 10.4–16.9 µm, were spherical in shape, and the protein content was 0.7–1.8 mg/g. After encapsulation by ionotropic gelation, beads ranged in size from 1620–1950 µm and were spherical in shape with a protein content of 1.0–2.5 mg/g. FTIR analysis indicated that both encapsulation processes were efficient. The cochleates encapsulated by ionotropic gelation were then tested for safety in pullets. No ill effect on the health of animals was observed upon physical or postmortem examination. In conclusion, this study was the first step in developing a potential oral *S.* Infantis vaccine safe for poultry using a novel cochleate encapsulation technology. Future studies are needed to determine the effectiveness of the vaccine.

## 1. Introduction

Chicken meat is the most widely consumed meat in the world, constituting 30% of global meat production [1]. Poultry products are accepted/marketed as healthier alternatives to red meat. Unfortunately, chicken meat is also an important reservoir for *Salmonella* spp. causing the foodborne disease salmonellosis in human beings. A significant increase in *Salmonella* has been observed in many countries over the past years, leading to international restrictions on imports and exports of chicken meat [2]. *Salmonella* is devastating to public health and has a negative economic impact on the poultry industry. In Europe, *Salmonella enterica* serovar Infantis (*S.* Infantis) is the most frequently reported serovar from chicken meat (36.5%) and broilers (56.7%) [3]. In the USA, *S.* Infantis is one of the most commonly isolated serotypes from sick humans and poultry meat products [4].

Since poultry products were identified as the major source of *Salmonella* infection, vaccination of poultry has become mandatory [5,6]. Most *Salmonella* control vaccines are parenteral [7,8]; however, the parenteral route has several disadvantages, such as the use of high bacterial concentrations for inoculation and the fact that vaccines require a lot of time for implementation and only generate humoral type immunity [5,6,7]. Oral vaccines of other *Salmonella* serotypes have been developed that use live or attenuated bacteria instead. The main disadvantage is the possibility of a mutation that leads to increased severity of disease or side effects [9]. For this reason, it is necessary to develop oral vaccines based on bacterial antigens, whose advantages are easy and quick application, reduction of animal stress, less handling and immunization of large numbers of animals in a short time, lower costs associated with the lower use of inputs and safer immunization [8,10]. 

An innovative strategy in the development of oral vaccines has been the cochleate system. Cochleates have been used to protect and deliver several bacterial compounds such as membranes, proteins and DNA as vaccines by the oral route [11,12]. Cochleates are phospholipid-calcium precipitates derived from the interaction of anionic lipid vesicles with divalent cations. They have a defined multilayered structure consisting of a solid, lipid bilayer sheet rolled up in a spiral [11]. The membrane from *S.* Infantis contains several negatively charged molecules, such as phospholipids, proteins and lipopolysaccharides (which can be used as a source of negatively charged components), to induce calcium-cochleate formation, as has been studied with other Gram-negative bacteria [11,12]. 

A disadvantage of cochleates is that they can very quickly release their contents at small intestine level because of their lipidic nature, which leads them to be degraded by enzymes and bile salts [13,14]. To improve the stability of the cochleates, we propose the use of encapsulation technology, which can protect different components of their intestinal degradation and release the contents in a controlled release form [11]. One of the most widely used techniques for the encapsulation of oral vaccines is spray drying [15,16] using maltodextrin/alginate mixtures as encapsulating materials that facilitate the generation of microparticles that can be carried in food or drinking water. This technique is economical and scalable [17]. Another successful methodology for this purpose is ionotropic gelation [18] using sodium alginate as an encapsulating material, which is a polymeric material that allows controlled release in the small intestine [19]. 

Currently, there is no evidence in the literature about the development of oral vaccines from *S.* Infantis cochleates. We hypothesize that it is possible to use *S.* Infantis membranes to develop cochleates and then encapsulate them by two different methodologies, generating safe vaccine formulations for poultry. The aim of this study was to develop and characterize *S.* Infantis cochleates protected by encapsulation technology as a potential vaccine and to determine its safety in pullets.

## 2. Materials and Methods 

### 2.1. Production of Cochleates from S. Infantis

#### 2.1.1. Isolation of *S.* Infantis

*S.* Infantis strains were obtained by microbiological isolation from cloacal swabs taken from chickens and layer hens belonging to commercial farms from the Valparaiso and Libertador General Bernardo O'Higgins regions, which are the major poultry production areas in Chile, using Cary Blair medium swabs. The torula was placed in a sterile test tube with 5 mL of Phosphate Buffered (PBS) (Sigma Aldrich, Merck®, Burlington, MA, USA) with novobiocin (20 ug/mL) (Sigma Aldrich, Merck®, Burlington, MA, USA) and incubated at 37 °C for 18 to 24 h. 

After incubation time 100 μL from each tube was spread on modified semisolid Rappaport Vassiliadis agar, (MSRV semisolid agar) (Oxoid®, Thermo Fisher, Waltham, Massachusetts, U.S.) smented with novobiocin (20 μg/mL) and incubated for 24 h at 41.5 ºC. Of the samples that presented suspicious growth, a roaster sample was taken and sowed by exhaustion in (Xylose lysine deoxycholate agar (XLD agar, Difco®, Merck®, Burlington, MA, USA); this sample was incubated at 37 ºC for 24 h. Samples that showed black or translucent colonies were isolated by seeding on XLD and McConkey agar, (Difco®, Merck®, Burlington, MA, USA). Once the colonies were isolated, a Polymerase Chain Reaction (PCR) was performed to confirm the genus by amplification of the *invA* gene [20]. The *Salmonella* spp. isolates were sent to the Chilean Institute of Public Health for serotyping using the Kauffman—White classification scheme. 

A bacterial sediment was prepared from *S.* Infantis in a bioreactor (New Brunswick bioflo 415, NJ, USA) with automatically controlled conditions (oxygenation of 40%, pH 7.0, temperature 37 °C and agitation 500 rpm) in order to obtain the highest yield of biomass. Th bacteria were centrifuged to obtain the bacterial sediment and washed with sterile Phosphate Buffered Water. The bacterial suspension was subjected to cold sonication pulses at 400 wats for 30 s using an Ultrasonic Processor UP400S sonicator (Hielscher Company, Berlin, Germany). 

#### 2.1.2. Bacterial Membrane Purification

For the purification of the bacterial membrane, bacterial sediment was suspended in a buffer containing 30 mmol/L of tris(hydroxymethyl)aminomethane (TRIS) at a rate of 1:10 volume of bacterial sediment per TRIS. This suspension was frozen at −80 °C for 2 h and defrosted by continuous sonication at 400 watts and 24 kHz (Hielscher Ultrasonic Processor UP400S; IU-P02, Berlin, Germany). The defrosted suspension was added to 1.5% (w/v) sodium deoxycholate (DOC) and was stirred at 150 rpm at 20 °C for 72 h and centrifuged at 250× *g* for 5 min and 15 °C. The supernatant was adjusted to a concentration of 5 mg soluble protein/mL by bicinchoninic acid method (BCA) (Protein Assay Kit, 71285, Merck®, Burlington, MA, USA).

#### 2.1.3. Cochleate Formation

**Figure 1:**
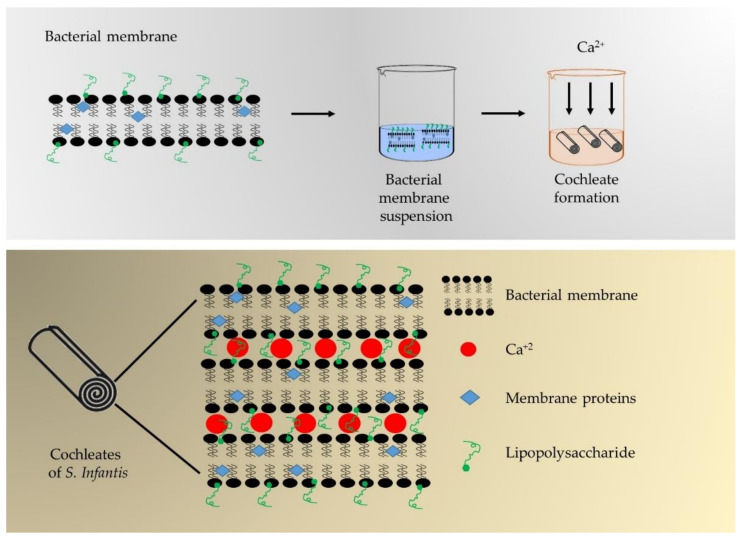
Simplified scheme of the procedure for the production of cochleates from *S.* Infantis and a graphic representation of the cochleates obtained.

Figure 1 shows a summary of the cochleate formation procedure. To induce calcium-cochleate formation, the bacterial membrane suspension was dripped (0.5 mL/min) into a cochleate formation solution (containing 0.363% w/v Tris, 0.584% w/v NaCl and 0.174% w/v CaCl_2_ at pH 10). Once the drops were administered, the dispersion was stirred for 3 h, and then a wash solution was added to remove the DOC (containing 0.121% w/v Tris and 0.087% w/v NaCl at pH 10). The solution was centrifuged at 2900× *g* for 40 min at 4 °C; the supernatant was discarded and the sediment reserved. The sediment was then re-suspended in the washing solution, and the soluble protein concentration was determined by BCA method.

#### 2.1.4. Cochleate Characterization

Cochleates were characterized by transmission electron microscopy (TEM). To obtain images by TEM, an aliquot of 10 µL of cochleate was taken and deposited on grids (300 Mesh Formvar/Carbon 50/pk, Bussines Electronics SPA, Legnano, Italy) and stained with 1% v/v aqueous uranyl acetate for 1 minute. Cochleates were observed in a transmission electron microscope (Philips Tecnai 12 BioTwin, FEI Company, Eindhoven, The Netherlands) operated at 80 kV. The photographs were processed through Megaview G2 Software (Soft Imaging System GmbH, Münster, Germany).

### 2.2. Cochleate Encapsulation

Cochleates were encapsulated by two different technologies: spray drying and ionotropic gelation. For spray drying, an encapsulating solution based on 0.5% w/v sodium alginate (Sigma-Aldrich, St. Louis, MI, USA) plus 20% w/v maltodextrin (20 dextrose equivalent, Licán Alimentos S.A., Chile) in distilled water was prepared. Cochleates were added to the encapsulating solution at 0% (as control, MP-0%), 5% (MP-5%), 10% (MP-10%) and 15% (MP-15%) v/v by magnetic agitation. The blends were then immediately fed to a B-290 Mini Spray Dryer (BÜCHI Labortechnik AG, Flawil, Switzerland). The inlet and outlet air temperatures were set at 140 ± 5 °C and 95 ± 5 °C, respectively. The air flow, rate of feeding and atomization pressure were 500 L/h, 8 mL/min and 20 psi, respectively.

For the ionotropic gelation method, sodium alginate (Sigma-Aldrich, St. Louis, MI, USA) was used as the encapsulating material. A sodium alginate solution (2% w/v in distilled water) was prepared and different concentrations at 0% (as control, B-0%), 5% (B-5%), 10% (B-10%) and 15% (B-15%) v/v of cochleates were added to this solution by magnetic agitation. The blends were then immediately fed to a B-390 Encapsulator (BÜCHI Labortechnik AG, Flawil, Switzerland) with particle diameter set to 1000 μm. Beads were formed by ionotropic gelation using a 5% w/v cross-linking solution of calcium chloride and then deposited in plastic boxes to be dried until reaching a constant weight at a temperature of 40 °C/6 h. Once dried, these beads were removed from their boxes and stored at room temperature.

### 2.3. Characterization of Encapsulated Cochleates

#### 2.3.1. Appearance and Color

Microparticles and beads were photographed using a Sony DSC–HX1 digital camera (Sony Corporation, Tokyo, Japan). Color was measured according to the Hunter Lab color scale (L*: lightness, a*: redness/greenness and b*: yellowness/blueness) with a Konica Minolta CR-300 (Konica Minolta Inc, Tokyo, Japan) colorimeter.

#### 2.3.2. Size

The particle size distribution was determined by laser diffraction using the Partica LA-960 Laser Scattering Particle Size Distribution Analyzer (HORIBA Scientific, Kyoto, Japan). Briefly, 0.5 g of the microparticles were placed in the powder jet dry feeder accessory with a pressure of 0.30 mPa. To prepare the bead samples, 1 g of beads were suspended in 10 mL of Milli-Q water and placed in a sample cell under magnetic stirring. During the measurement, a 650 nm laser diode passed through the particle suspension; the scattered light was detected and collected by a silicon photo diode detector. All the samples were analyzed in triplicate at 25 °C.

#### 2.3.3. Morphology

The microparticles and beads were observed by Scanning Electron Microscopy (SEM). The dried samples were mounted on a cylindrical aluminum stub using double–sided tape to sputter coat them with gold (twice) at 20 kV in an argon atmosphere by a PELCO 91,000 sputter coater unit (Ted Pella, Inc., Redding, CA, USA). Coated samples were then examined by an SEM (LEO, 1420 VP, Cambridge, UK) equipped with an energy dispersive X-ray spectroscopy (EDS) using an accelerating voltage of 25 kV.

#### 2.3.4. Protein Content

The protein content of microparticles and beads was determined using a modification of the micro-Kjeldahl method (AOAC, method 960.52, N × 6.25). Briefly, aliquots of 1 g of sample were digested with 500 mg K_2_SO_4_ and 2.0 mL of CuSO_4_/H_2_SO_4_. The digestion procedure begins at a temperature of 200 °C which then reaches 400 °C. A sample of 2.0 mL of water was also digested simultaneously as the blank. The samples and blank digests were then alkalinized with NaOH and steam distilled. Released ammonia was absorbed in solutions of 2% boric acid and nitrogen content was determined by titration with 10 mM HCl after blank correction.

#### 2.3.5. Fourier Transform Infrared Spectroscopy (FTIR)

FTIR analyses were performed on cochleates, microparticles and beads. An ATR/FTIR interspect 200-X spectrometer (Interspectrum OU, Tartu maakond, Estonia) provided FTIR spectra for each sample. Spectroscopic measurements were performed directly using the PIKE Miracle TM accessory in a Ge single reflection crystal plate. A concave tip was used for all FTIR spectra. An average of 20 scans over the spectral range of 600 to 4000 cm^−1^ yielded each spectrum.

### 2.4. In Vivo Safety Trial

All protocols were approved by the Bioethics Committee at the University of Chile (Certificate of Bioethics 2018-18186-VET-UCH). In order to accomplish the committee requirements, all personnel involved in poultry management demonstrated appropriate skills through professional/technical degrees and previous experience. Fourteen one-day-old female Rhode Island pullets received from a commercial hatchery were equally divided into two groups; they were treated with cochleates encapsulated by ionotropic gelation at 0% (B-0% as control group) and 15% (B-15% as treatment group). The birds were kept under strict biosecurity conditions at the Avian Pathology Laboratory facilities for six weeks in two community cages inside two separated isolated rooms; then, they were vaccinated with cochleates encapsulated by ionotropic gelation. Commercial pullet growth diet, formulated according to nutritional requirements proposed by the National Research Council [21], and water ad libitum were offered throughout the experiment.

The beads measured 1950 ± 28 µm, and each dose was prepared in individual Eorf tubes. The pullets’ beaks were carefully opened, and the beads were given; between five and six beads were administered reach the calculated dose. Then, 0.5 mL of PBS was given with a tuberculin syringe to assure bead consumption. Over 5 days, the treatment group was vaccinated with 65 µg cochleates to evaluate the safety of the formulation. The control group was treated with empty alginate beads. During the experiment, all birds were examined daily by the same person for clinical signs according to the parameters proposed by Morton and Griffiths with some modifications [22]; the temperature was taken intra-cloacally with a digital thermometer, while cardiac and respiratory frequencies were measured with a stethoscope. The parameters and scoring are shown in Table 1. A score was assigned to each parameter and summed for a total score for each animal. If a total score ranging from 5–9 was given, the bird would be observed twice a day; a range of 10–14 could result in possible euthanasia, and a total score over 15 would lead to the immediate termination of the experiment and a refining of the protocol. 

At the end of the safety study, the birds were euthanized by cervical luxation and immediately necropsied to look for pathological changes. Any unspecific signs in the gastrointestinal tract that could be caused by the vaccine formulation, such as petechial, hyperemia, hemorrhages, inflammation, ulcers and necrosis, were searched for. The birds were examined from tongue to rectum, and other organs (spleen, lungs, liver, kidneys, and heart) were inspected. 

### 2.5. Statistical Analysis

After verifying the normality of the data with a Shapiro–Wilk test, the characteristics of beads and microparticles were analyzed by ANOVA and a Tukey test (*p* < 0.05). All statistical analyses were run with Statistix 8 software (Analytical Software 2003; Tallahassee, FL, USA). Data is presented as average ± standard deviation. The safety trial provided group scores, and thus, the data is analyzed as descriptive data. 

## 3. Results

### 3.1. Cochleate Development

**Figure 2:**
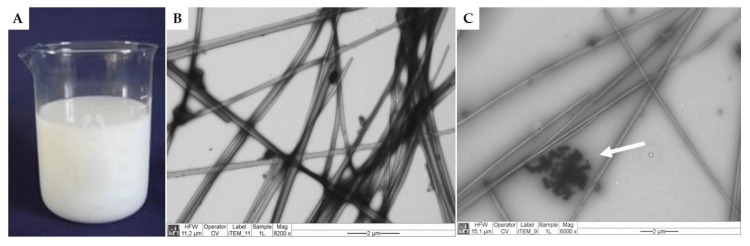
Macroscopic (**A**) and microscopic aspect by transmission electron microscopy of cochleates derived from *S*. Infantis (**B** and **C**). The white arrow indicates crystals from the calcium solution added to the formulations for cochleate formation (**C**).

Milky-white colorless suspensions of cochleates (Figure 2A) were obtained with a soluble protein content ranging from 1.0 to 2.1 mg/mL. Figure 2B and C show a micrograph of the cochleates from *S.* Infantis observed by TEM. The cochleates had a tubular structure with widths ranging from 0.1 to 0.2 µm and variable lengths. Figure 2C shows calcium crystals (indicated with a white arrow) typical of solutions used to form cochleates. Figure 1 outlines the possible arrangement of the cochleates, where the membranes of *S.* Infantis are arranged in an orderly manner bound by calcium atoms and rolled up on themselves.

### 3.2. Cochleate Encapsulation and Characterization

**Figure 3:**
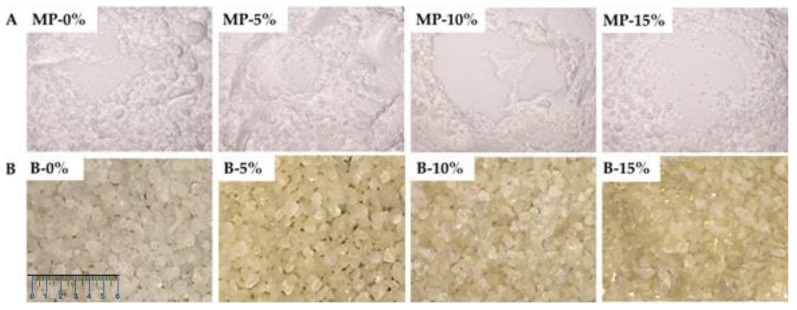
Macroscopic view of microparticles (MP) **(A)** and beads **(B)** at different concentrations of cochleates of *S.* Infantis (0–15% v/v).

In order to obtain viscosity values compatible with spray drying (microparticles) and ionotropic gelation (beads) methods, different concentrations of cochleates and encapsulant material were tested. For the microparticles, we used 0–15% v/v cochleates, 0.5% w/v alginate and 20% w/v maltodextrin. For the beads, we used 0–15% v/v cochleates and 2% w/v alginate. Figure 3 shows the microparticles and beads obtained by spray drying and ionotropic gelation methods, respectively. The microparticles were white powders (Figure 3A) that did not differ between formulations (Table 2). The control beads had a spherical shape that changed as the concentration of cochleates increased. The beads had heterogeneous and elongated shapes with irregular borders for the highest concentrations of cochleates at 10 (B-10%) and 15% (B-15%) (Figure 3B). The control beads were transparent. The beads encapsulating the cochleates were light brown with significant differences in all color parameters compared to the controls, but they did not differ significantly between formulations (Table 2). The beads were significantly larger than the microparticles (Table 2). The beads ranged from 1,620 to 1,950 μm; meanwhile, the microparticles ranged from 10–17 μm. For the beads, size increased as the cochleate concentration increased. For the microparticles, MP-15% was the same size as the control (MP-0%) and smaller than MP-5% and MP-10%.

**Figure 4:**
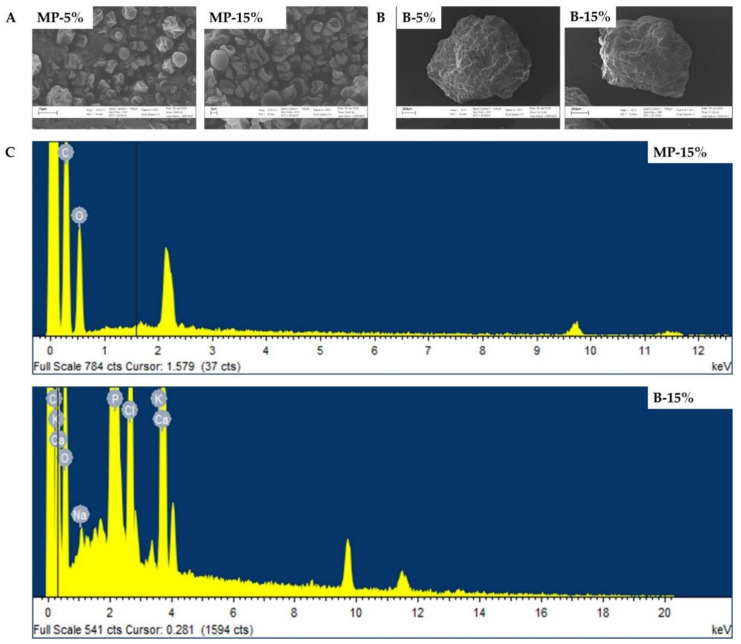
Surface morphology by scanning electron microscopy of microparticles at 5% (MP-5%) and 15% (MP-15%) cochleate of *S*. Infantis (**A**) and beads at 5% (B-5%) and 15% (B-15%) of cochleates of *S*. Infanti*s* (**B**). Elemental composition by energy dispersive X-ray spectroscopy of microparticles (MP) and beads (**B**) at 15% of cochleates of *S*. Infantis (**C**).

The surface morphological characterization by scanning electron microscopy (SEM) and elemental composition by energy-dispersive X-ray spectroscopy (EDS) of selected microparticles (M-5% and MP-15%) and beads (B-5% and B-15%) are presented in Figure 4. After the cochleates’ encapsulation by spray drying, microparticles with spheroidal shapes and dented surfaces were observed (Figure 4A). There were no morphological differences between different concentrations of cochleates in the microparticles. 

Electronic micrographs showed well-formed beads with spheroidal shapes and heterogeneous surfaces except for B-15%, where elongated beads with sharp edges were observed (Figure 4B). EDS analysis indicates that the encapsulation was efficient since only atoms typical of the chemical composition of the encapsulating materials were found, such as oxygen (O) and carbon (C) in the microparticles, which are the main components of maltodextrin and alginate. For the beads, several atoms typical of sodium alginate (C, O, Na), calcium (Ca) and chlorine (Cl) from the calcium chloride solution used for crosslinking were found (Figure 4C). 

The protein content in the microparticles and beads is presented in Table 2. No protein content was detected in the control microparticles. The protein concentration in the microparticles and beads loaded with cochleates increased significantly with increasing cochleate concentration. When comparing the two encapsulation methods, it was observed that the beads were able to retain a greater amount of protein, reaching 2.5 mg/g in B-15% compared to 1.8 mg/g in MP-15%, indicating that the beads retain 28% more protein than the microparticles.

**Figure 5:**
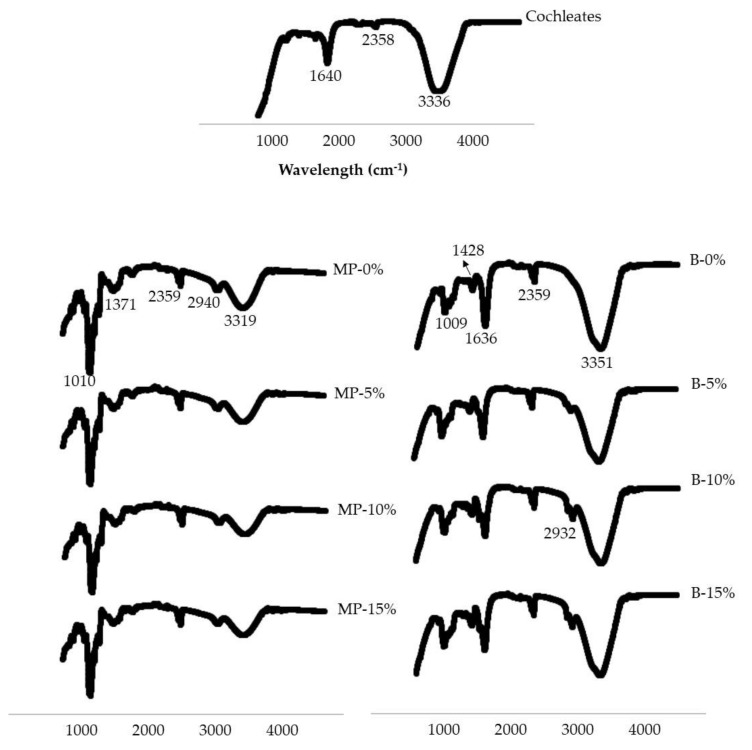
FTIR of cochleates of *S*. Infantis, microparticles (MP) and beads (B) with different concentrations of cochleates of *S*. Infantis (0–15% v/v).

Figure 5 shows the FTIR of the cochleates, microparticles and beads. In the case of cochleates, the most important bands observed in the FTIR are found at 3336 cm^−1^, corresponding to O-H bonds. The second most important band corresponds to N-H bending bonds (1640 cm^−1^). For the microparticles an important band characteristic of O-H stretching bonds was observed (3319 cm^−1^). Then, less important bands were observed at 2940 cm^−1^ and 2359 cm^−1^, corresponding to C-H stretching bonds. Subsequently, bands characteristic of C-O stretching bonds were observed (1371 cm^−1^ and at 1010 cm^−1^). All of these bands are related to the alginate and maltodextrin molecular structures. 

The FTIR of the beads has a characteristic broad band of O-H stretching bonds (3351 cm^−1^). Then, bands of the asymmetric and symmetric bond of the carboxylate group C=O of alginate (1636 cm^−1^ and 1428 cm^−1^) were observed. Finally, a band representing the C-O stretching bonds (1009 cm^−1^) was observed.

### 3.3. In Vivo Safety Trial

The physical condition of the birds was evaluated whenever contact was made for husbandry purposes. The birds were alert to the environment and to manipulation and were active at a level expected for their age. According to the scoring system described in Table 1, no altered parameters were observed in the control or treatment group. Fifteen days after vaccination, the euthanization was carried out. In the necropsy studies, neither macroscopic nor pathological lesions were found in the tissues and organs evaluated, which all showed a physiological aspect in their surface and parenchyma. Thus, no differences were observed between the two groups. Tongue, esophagus, crop, proventriculus, gizzard and small and large intestines did not exhibit any change; neither did the main organs. The intestinal lumen was evaluated for changes in its wall integrity or mucosal face, but no lesions were found.

## 4. Discussion

The milky appearance of *S.* Infantis cochleates is explained by the formation of a colloidal suspension of bacterial membranes dripped into a calcium chloride solution. This process is known as the trapping method and involves the rolling up of a negatively charged compound (lipids, proteins and lipopolysaccharides) present in bacterial membranes through interaction with positively charged multivalent ions (Ca^+2^), minimizing their interaction with water and promoting the formation of tubular structures [11,14]. The tubular appearance of the cochleates observed in Figure 1 B and C is explained by the fact that they are formed by continuous lipid lamellae that are rolled on themselves in a spiral structure [12]. It has been proposed that the positively charged calcium atoms (Ca^+2^) interact ionotropically with the negatively charged membranes, acting as a bonding agent that generates the rolling of the membranes [11]. This provides stability to the cochleates, to be used as vaccine prototypes, since the antigenic structures are protected inside the cochleate structure [11]. 

In this study we encapsulated the cochleates to provide them greater stability for their passage through the gastrointestinal tract [23]. The technologies and materials selected to encapsulate the cochleates were chosen based on their scalability and low cost, which are very important properties for veterinary applications. Both spray drying and ionotropic gelation can encapsulate a high amount of liquid material [24,25], such as cochleate suspensions. Moreover, microparticles and beads produced with the proposed ingredients protect and release their contents in the small intestine in a controlled manner as described in other applications [19,26]. The encapsulation of the cochleates by the proposed methodologies was successful, encapsulating high concentrations of cochleates, up to 15% v/v. The encapsulating materials used (maltodextrin and alginate powder) for the microparticles are white, similar to the cochleate suspensions; therefore, it was expected that the microparticles would be white. Other studies using these encapsulant materials have reported that the microparticles turn the same color of the core material but with lighter shades, depending on the concentrations of the encapsulant materials and the core material. For example, Churio et al. [27] encapsulated ferrous sulfate (green color) as a sment for pigs with maltodextrin by spray drying, obtaining light green microparticles. Alginate beads are commonly translucent and turn the color of the material they encapsulate [28]. This explains the change in color of the cochleate encapsulating beads.

A change in the shape of the beads was observed as the concentration of cochleates increased (Figure 3B and Figure 4B), varying from spherical shapes to more flattened shapes with irregular edges. Shape changes may be the result of the addition of liquid cochleates to the bead-forming mixture, reducing the viscosity of the solutions, altering the fluidity of the dripping process and deforming the drops that fall into the crosslinking solution [29,30]. 

The shape of the microparticles was observed in detail by scanning electron microscopy and was considered to be typical morphology for maltodextrin/alginate microparticles produced by spray drying. Invaginations resulting in folds and protrusions occur during the drying process [31,32].

Due to the encapsulation method, the sizes of the beads are significantly bigger than the microparticles. As expected, we observed a clear tendency of increased sizes in both systems, beads and microparticles, with an increase in cochleate concentration. Unexpectedly, MP-15% produced smaller microparticles than lower concentrations (MP-5% and MP-10%). Interestingly, other authors have reported that the final particle size, using spray drying technology, is affected by the concentration of the feed solution “but in a non-linear way” [33]. For instance, Elversson et al. [34] developed lactose microparticles, observing that particle size increased substantially between 1–5% w/w; however, the increase was reduced at concentrations above 5% w/w. This behaviour was attributed to lower yields at higher feed concentrations, by differences in the effective particle density and also differences in drying rate.

The success of the encapsulation process is demonstrated by the protein content, FTIR and EDS analysis. Protein content is a very important estimate because it is related to the amount of antigens contained in the formulations. As expected, protein concentration was higher in formulations with higher cochleate loads. Interestingly, the beads were able to retain more protein than the microparticles. This could be explained by the fact that ionotropic gelation allows a high retention of the encapsulated compound. This process takes place when a bonding zone is produced between an alginate G-block that is electrostatically bonded to another G-block through the calcium ions, forming a structure known as the "egg box" model, which protects the encapsulated compounds with high efficiency [35,36].

The FTIR analysis shows the different interactions that occurred between the cochleates and the encapsulant materials to form the microparticles and beads. In the FTIR analysis of the beads, the main interaction was O-H bonds. These interactions may be the results of hydration of the hydrophilic groups of the alginate, electrostatic interactions between alginate and some groups of the cochleates (such as amino acids) and/or the formation of bonds with adjacent hydroxyl groups [28]. The FTIR spectrum of the beads shows the characteristic peaks of alginate but with a small shift, proving successful crosslinking of the alginate carboxylate group with Ca^+2^ [37,38]. FTIR analysis of the microparticles showed characteristic peaks of alginate and maltodextrin molecular structures, which are the major constituents. Both beads and microparticles loaded with cochleates have similar FTIR spectra as the control samples (MP-0% and B-0%). Since the cochleates are protected by the encapsulating materials (alginate/maltodextrin for microparticles and alginate for the beads), the similarity between the spectrums is indicative of successful encapsulation. This agrees with the results obtained by Shuddhodana et al. who encapsulated artemisinin cochleates with alginate [37]. The presence of the same bands in the FTIR spectral analyses as those in the encapsulating material and in the encapsulating material plus cochleate confirmed the association of their constituents and suggested efficient encapsulation.

No nitrogen atoms, typical of protein and indicative of cochleates, were observed by EDS analysis of the surface of the microparticles (MP-15%) and beads (B-15%), confirming an efficient encapsulation of the cochleates by both technologies.

After the development of novel vaccine prototypes, *in vivo* safety trials were carried out. In this study, we tested an oral vaccine prototype with antigens obtained from bacterial membranes, which is different from most effective oral vaccines that are made with live or attenuated agents [39]. The choice of the oral route was based on the large number of animals in poultry farms (broilers and layers), where parenteral vaccination is impossible when birds are in production; furthermore, intramuscular vaccination in broilers is not viable because of it would affect the final commercial product, i.e., meat. Oral vaccines can be administered through water or food [40], reducing the cost and biosecurity risks associated with contact with workers. Moreover, in the case of *S. Infantis* where infection is mainly oral, the development of a vaccine given orally is highly desirable.

The vaccine developed by ionotropic gelation, which has a higher retention of the antigens, was tested orally at the maximum concentration in pullets. The safety trial results showed that the novel *S. Infantis* vaccine prototype is harmless to birds: no physical changes or pathological alterations were found in any segment of the digestive tract or any organ at the necropsy. The next step in this research will be to measure the immune response in the birds and conduct a challenge study against *S. Infantis* to determine the effectiveness of the vaccine against this pathogen.

## 5. Conclusions

In this study, we derived cochleates from the bacterial membranes of *S.* Infantis and then efficiently encapsulated the cochleates using two different technologies, spray drying and ionotropic gelation, to develop an oral vaccine prototype. Encapsulation by ionotropic gelation was more efficient, obtaining millimeter-sized beads (1620–1950 µm) with a protein content between 1.0–2.5 mg/g (reflecting the high content of antigens). Beads containing 15% v/v cochleates were delivered orally to birds for a safety test. During the safety study, no physical or health alterations were observed in the birds. In the post-mortem examination, no pathological alterations or lesions were observed in the gastrointestinal tract or in the organs analyzed. In this study, we took the first step toward the development of a novel prototype oral vaccine against *S. Infantis*. It is essential to analyze the immune response of the birds and the efficiency of the vaccine in future studies.

## Figures and Tables

**Table 1:** Parameters evaluated in the safety trial.

Parameters	Signs	Score
Weight loss	No alterations Weight loss below 10% Weight loss between 10–20% Weight loss greater than 20%	0 1 2 3
Aspect	No alterations Ruffled feathers Ruffled feathers + wings and tail dropped Ruffled feathers + wings and tail dropped + dirty tail	0 1 2 3
Behavior	No alterations Feeding activity decreased (observed at feeding time) Careless of environment, sagging through their legs to sitting position Depressed birds—birds with stupor	0 1 2 3
Vital signs	No alterations Increment until 2 °C in temperature Increment greater than 2 °C in temperature Previous signs + change in cardiac and respiratory frequency	0 1 2 3

**Table 2:** Characteristics of microparticles (MP) and beads (B) with different concentrations of cochleates of *S.* Infantis (0–15% v/v).

**Parameters**	**Microparticles**			
	**MP-0%**	**MP-5%**	**MP-10%**	**MP-15%**
Color				
L*	91.5 ± 1.2^a^	90.3 ± 0.9^a^	89.1 ± 1.1^a^	89.6 ± 1.3^a^
a*	-1.1 ± 0.2^a^	-1.2 ± 0.4^a^	-0.9 ± 0.2^a^	-1.2 ± 0.4^a^
b*	1.7 ± 0.3^a^	1.8 ± 0.4^a^	1.5 ± 0.2^a^	1.6 ± 0.4^a^
Size (µm)	10.4 ± 0.5	15.8 ± 0.9	16.9 ± 0.5	10.6 ± 0.1
Protein content (mg/g)	n.d	0.7 ± 0.3^a^	1.4 ± 0.2^b^	1.8 ± 0.3^c^
	**Beads**			
	B-0%	B-5%	B-10%	B-15%
Color				
L*	33.4 ± 1.5^a^	28.1 ± 1.8^b^	27.8 ± 1.3^b^	29.2 ± 1.1^b^
a*	1.3 ± 0.5^a^	2.5 ± 0.3^b^	2.4 ± 0.7^b^	2.6 ± 0.3^b^
b*	1.7 ± 0.4^a^	4.6 ± 0.4^b^	4.7 ± 0.8^b^	4.8 ± 0.6^b^
Size (µm)	1,680 ± 82^a^	1,620 ± 36^a^	1,700 ± 18^a^	1,950 ± 28^b^
Protein content (mg/g)	n.d	1.0 ± 0.2^a^	1.7 ± 0.4^b^	2.5 ± 0.3^c^

Different letters indicate significant differences (a and b, p < 0.05). n.d: not detected. Color parameters were determined by the Hunter Lab color scale (L*: lightness, a*: redness/greenness and b*: yellowness/blueness).
